# Real-world case series: Birch triterpenes gel efficacy in 2 patients with junctional epidermolysis bullosa

**DOI:** 10.1016/j.jdcr.2025.03.020

**Published:** 2025-04-04

**Authors:** Alexia Collins, Elisha M. Myers, Kristina M. Derrick, Sharon A. Glick

**Affiliations:** a Department of Dermatology, SUNY Downstate Health Sciences University, Brooklyn, New York; b Department of Dermatology, New York City Health & Hospitals Corporation - Kings County Hospital, Brooklyn, New York; c Department of Dermatology, Charles E. Schmidt College of Medicine, Florida Atlantic University, Boca Raton, Florida; d Global Health, Trinity College Dublin, Dublin, Ireland

**Keywords:** Birch triterpenes gel, Dystrophic Epidermolysis Bullosa (DEB), epidermolysis bullosa, filsuvez, junctional epidermolysis bullosa (JEB), oleogel

## Introduction

Epidermolysis bullosa (EB) is a group of inherited disorders characterized by fragile skin that blisters with minimal trauma.^1^ Among the 4 major subtypes, one of the rarest is junctional EB (JEB), a disease where blisters form in the lamina lucida due to fragile skin.^2^ JEB is an autosomal recessive disorder caused by mutations in genes encoding laminin 332, type XVII collagen, integrin α6β4, and integrin α3 subunit proteins.^3^ These mutations disrupt cell adhesion, leading to symptoms.

Patients with JEB are classified into 2 categories: generalized severe and generalized intermediate.^2^ Both the severe and intermediate forms present with blisters and erosions that appear in the neonatal period.^2^ Management has traditionally focused on supportive care. In 2023, birch triterpenes gel (Filsuvez) became the first Food and Drug Administration (FDA)-approved medication for JEB.^4^ Despite this, clinical trials showed birch triterpenes gel was not significantly more effective than placebo in promoting wound closure.^5^ The available data on the efficacy of birch triterpenes gel for treating patients with JEB are limited compared to its data for dystrophic EB.^5^ This limitation arises because only 26 of 223 participants in the clinical trial had JEB, whereas the majority had dystrophic EB.^5^ Because of the small number of patients with JEB included, the subgroup analysis did not show a significant difference between birch triterpenes gel and placebo in the proportion of patients achieving their first complete wound closure within 45 or 90 days.^5^ This small sample size affects the ability to detect meaningful differences, potentially underestimating birch triterpenes’ gel benefits for JEB.

Real-world evidence is essential for assessing the true effectiveness of birch triterpenes gel in JEB. Data from everyday clinical practice can provide valuable insights into its impact on wound healing and patient outcomes, supporting its acceptance and insurance coverage when clinical trial data may not. We present 2 pediatric patients with severe, refractory JEB successfully treated with birch triterpenes gel.

## Case 1

A 12-year-old girl with biopsy-proven JEB linked to a homozygous LAMB3 gene variant presented for a follow-up of 6 months after being prescribed birch triterpenes gel for her JEB.^6^ She had previously used triamcinolone, mupirocin, petrolatum, gentian violet, gentamicin, and Mepilex dressings with minimal improvement. Because of the severity of her wounds, birch triterpenes gel was obtained before FDA approval through an institutional review board-approved protocol for expanded access. She applied birch triterpenes gel daily to all lesions (left ear, right axilla, chest, right ear, nails, buttocks, and thighs). At 6 months, she reported improvement in her left ear and right axilla lesions, with no other significant wound changes. She no longer needed dressing changes for her left ear and a reduced number of dressing changes for the right axilla. The patient denied systemic symptoms, including fever and infection, and continued using birch triterpenes gel during an active cellulitis infection of the ears, despite being contraindicated.

**Fig 1:**
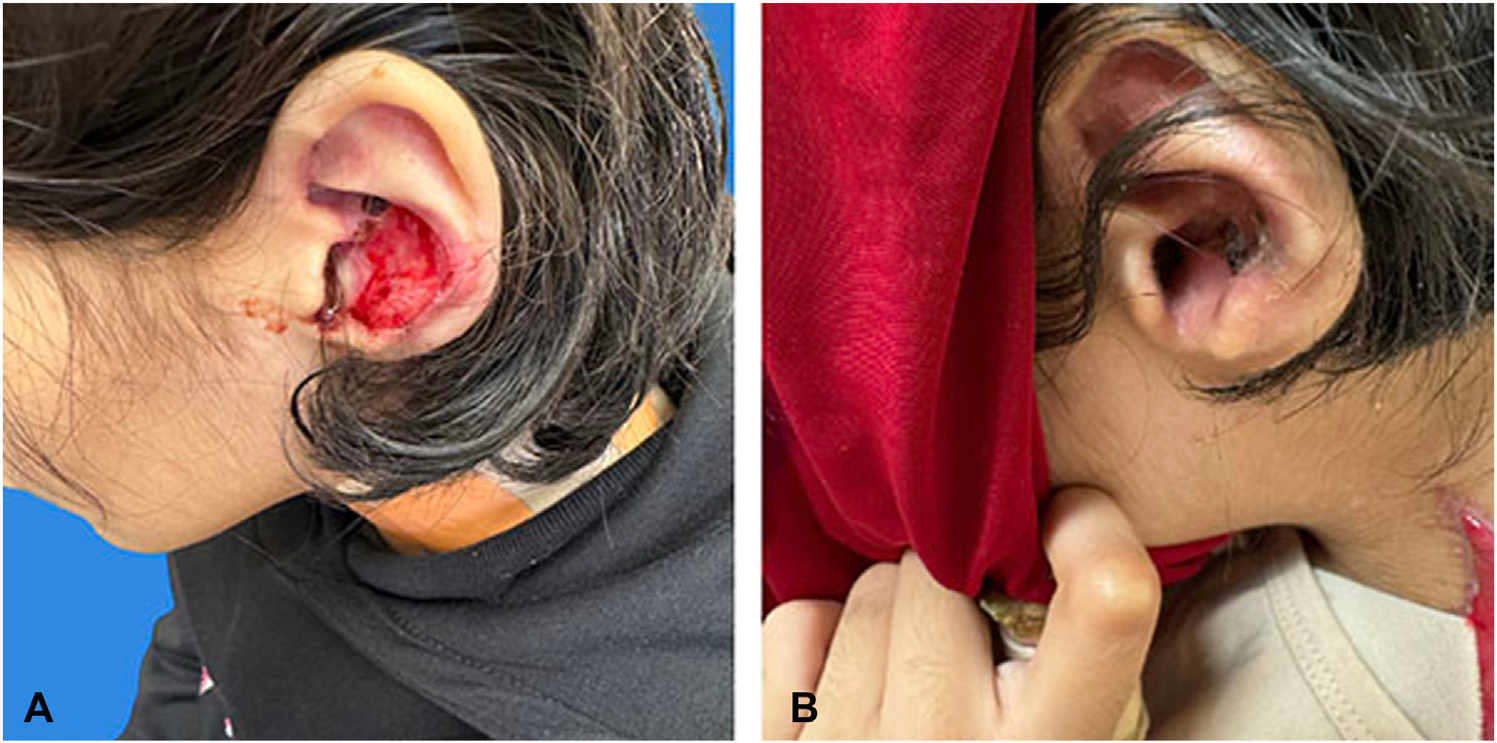
**(A)** Patient 1: The left ear prior to Filsuvez use, showing extensive erosions and granulation tissue before treatment initiation. **(B)** The left ear after 6 months of Filsuvez use, demonstrating significant improvement with reduced erosions and healing epithelialized skin following treatment.

**Fig 2:**
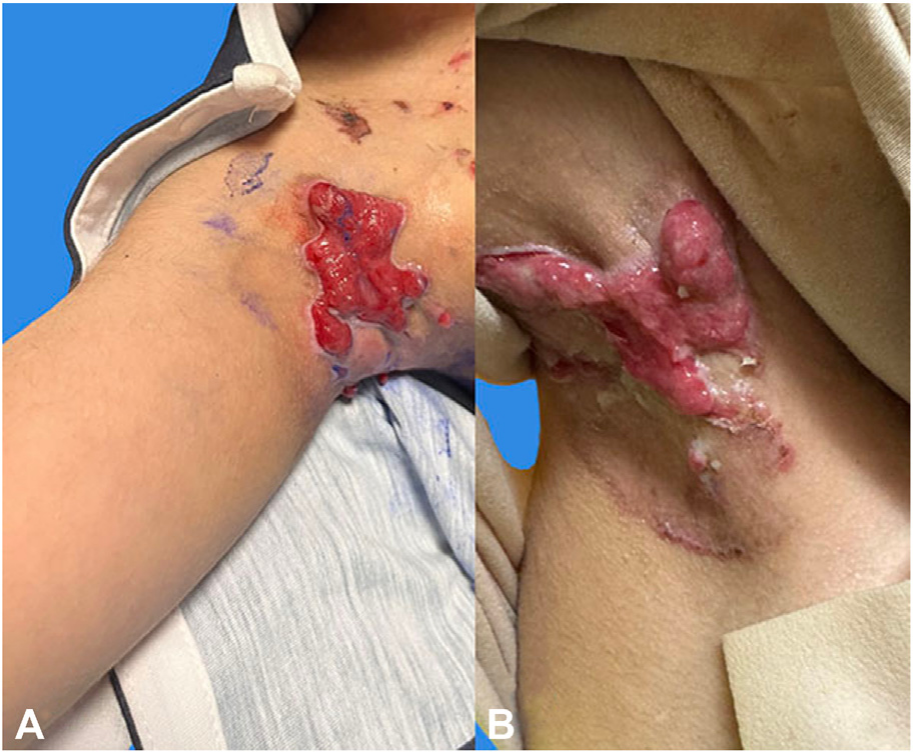
**(A)** Patient 1: Baseline appearance of the left axilla, showing active ulceration and inflammation. **(B)** The left axilla after 6 months of birch triterpenes gel use, showing significant granulation tissue formation and partial wound healing after application of birch triterpenes gel.

Physical examination showed several skin erosions and granulation tissue, including hemorrhagic crusting on the left conchal bowl, crusting and erosions on the right conchal bowl, and large eroded plaques on the neck, chest, and axilla. The left ear (Fig 1) and right axilla (Fig 2) wounds improved significantly with epithelialization and reduced wound size, although granulation tissue persisted in other areas.

## Case 2

**Fig. 3:**
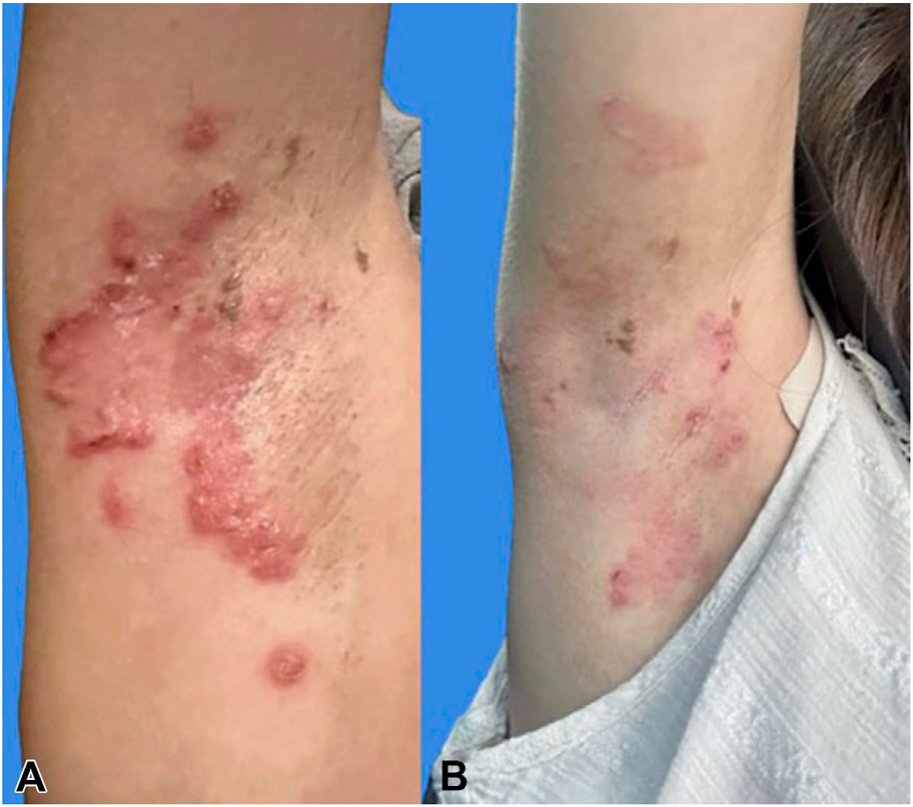
**(A)** Right axilla before treatment with birch triterpenes gel, showing multiple erythematous lesions, some with signs of excoriation and active inflammation. Areas of hyperpigmentation and localized swelling also are seen. **(B)** Right axilla 1 month after treatment with birch triterpenes gel. There is a noticeable reduction in the severity of inflammation and erythema. Some lesions appear to be healing, with less excoriation and reduced swelling, although areas of hyperpigmentation persist.

A 19-year-old woman with JEB generalized intermediate, diagnosed 10 years ago via genetic testing showing a homozygous autosomal recessive mutation in the LAMB3 gene, presented with blistering in friction-prone areas such as the axillae, neck, lower legs, and groin, along with fissures on her feet. Prior treatments included nonadherent dressings, gentamicin ointment, CeraVe moisturizer, and petrolatum. She was started on birch triterpenes gel through the Chiesi Total Care program after her insurance denied coverage, citing a lack of efficacy for the JEB subtype. After 1 month of treatment, she reported improvement in erosions on her right axilla and lower legs. She alternated between gentamicin and birch triterpenes gel for wound care. She struggled to keep dressings adhered to the axilla but applied birch triterpenes gel and dressings at night, removing them in the morning, while absorbent foam dressings on her legs stayed on continuously. She changed dressings and reapplied birch triterpenes gel daily. Her wound size and severity have reduced (Fig 3) without pain, pruritus, or infection. The patient no longer needs daily dressing changes. As she continues to improve with birch triterpenes gel, we are pursuing appeals for insurance coverage.

## Discussion

Herein, we describe 2 patients treated with birch triterpenes gel for JEB, showing significant improvement in wound size, healing, pain reduction, and overall quality of life. Despite limited efficacy data, birch triterpenes gel allowed for less frequent dressing changes, reduced analgesic use, and decreased burden on patients and caregivers. birch triterpenes gel were well-tolerated, with no adverse effects, even in cases of active wound infection.

Although the efficacy and safety of Oleogel-S10 (birch triterpenes) for epidermolysis bullosa clinical trial data did not demonstrate a significant difference between birch triterpenes gel and placebo in achieving wound closure for patients with JEB, this outcome could be attributed to the small sample size of the JEB subgroup (only 26 of 223 participants) and the inherent difficulty of detecting clinically significant differences with such a small sample size not powered for such subgroup analysis.^5^ Although in only 2 cases, our patients demonstrated that birch triterpenes gel may still provide substantial clinical benefits for patients with JEB. Unfortunately, because of the results from the clinical trial, one of our patients has had tremendous difficulty obtaining insurance approval for birch triterpenes gel. According to GoodRx, the price of a 23.4-g tube of birch triterpenes gel is $1850 USD, making it financially inaccessible to most patients whose insurance companies deny approval.^7^ Given that birch triterpenes gel remains the only FDA-approved treatment for JEB, improved access to birch triterpenes gel could dramatically improve patient outcomes, particularly in severe cases such as those presented here. To achieve this, it is crucial for physicians to actively report their real-world experience using birch triterpenes gel for JEB.

In terms of safety, birch triterpenes gel was well-tolerated in both of our cases with no serious adverse effects, including when used outside the FDA guidelines during an active purulent cellulitis. Further studies are needed to fully understand the safety profile of this medication. Reports such as ours can provide valuable evidence that may influence clinical guidelines leading to improved insurance coverage and provide data on the safety and efficacy of birch triterpenes gel.

## Conflict of interests

Dr Derrick has participated in the Chiesi advisory board meeting and served as a speaker. Amryt Pharmaceuticals provided medication through an expanded access program to both patients. Dr Glick is a member of the Epidermolysis Bullosa Clinical Characterization and Outcomes Database. Dr Glick was an investigator and Dr Derrick was a subinvestigator for a clinical trial with Lenus Therapeutics for an EB treatment.
